# Evaluation of the genetic polymorphism of *Plasmodium falciparum *P126 protein (SERA or SERP) and its influence on naturally acquired specific antibody responses in malaria-infected individuals living in the Brazilian Amazon

**DOI:** 10.1186/1475-2875-7-144

**Published:** 2008-07-30

**Authors:** Lilian Rose Pratt-Riccio, Selma Sallenave-Sales, Joseli de Oliveira-Ferreira, Bruno T da Silva, Monick Lindenmeyer Guimarães, Fátima Santos, Thatiane S de Simone, Mariza G Morgado, Salvatore G de Simone, Maria de Fátima Ferreira-Da-Cruz, Cláudio T Daniel-Ribeiro, Mariano G Zalis, Daniel Camus, Dalma M Banic

**Affiliations:** 1 Laboratório de Pesquisas em Malária, Departamento de Imunologia, Instituto Oswaldo Cruz, Fiocruz, Avenida Brasil 4365, Manguinhos, Rio de Janeiro, Brazil; 2 Laboratório de AIDS e Imunologia Molecular, Departamento de Imunologia, IOC, Fiocruz, Rio de Janeiro, Brazil; 3 Departamento de Entomologia, LACEN, Porto Velho, Rondônia, Brazil; 4 Laboratório de Bioquímica de Proteínas e Peptídeos, IOC, Fiocruz, Brazil; 5 Hospital Universitário Clementino Fraga Filho, Universidade Federal do Rio de Janeiro, Rio de Janeiro, Brazil; 6 Service de Parasitologie-Mycologie, Faculte de Médecine, Lille, France

## Abstract

**Background:**

The *Plasmodium falciparum *P126 protein is an asexual blood-stage malaria vaccine candidate antigen. Antibodies against P126 are able to inhibit parasite growth *in vitro*, and a major parasite-inhibitory epitope has been recently mapped to its 47 kDa N-terminal extremity (octamer repeat domain – OR domain). The OR domain basically consists of six octamer units, but variation in the sequence and number of repeat units may appear in different alleles. The aim of the present study was to investigate the polymorphism of P126 N-terminal region OR domain in *P. falciparum *isolates from two Brazilian malaria endemic areas and its impact on anti-OR naturally acquired antibodies.

**Methods:**

The study was carried out in two villages, Candeias do Jamari (Rondonia state) and Peixoto de Azevedo (Mato Grosso state), both located in the south-western part of the Amazon region. The repetitive region of the gene encoding the P126 antigen was PCR amplified and sequenced with the di-deoxy chain termination procedure. The antibody response was evaluated by ELISA with the Nt47 synthetic peptide corresponding to the P126 OR-II domain.

**Results:**

Only two types of OR fragments were identified in the studied areas, one of 175 bp (OR-I) and other of 199 bp (OR-II). A predominance of the OR-II fragment was observed in Candeias do Jamari whereas in Peixoto de Azevedo both fragments OR-I and OR-II were frequent as well as mixed infection (both fragments simultaneously) reported here for the first time. Comparing the DNA sequencing of OR-I and OR-II fragments, there was a high conservation among predicted amino acid sequences of the P126 N-terminal extremity. Data of immune response demonstrated that the OR domain is highly immunogenic in natural conditions of exposure and that the polymorphism of the OR domain does not apparently influence the specific immune response.

**Conclusion:**

These findings confirm a limited genetic polymorphism of the P126 OR domain in *P. falciparum *isolates and that this limited genetic polymorphism does not seem to influence the development of a specific humoral immune response to P126 and its immunogenicity in the studied population.

## Background

The *Plasmodium falciparum *P126 protein is an asexual blood-stage malaria vaccine candidate antigen. P126, also referred to as SERA [1] and SERP [2], is synthesized during the late trophozoite and schizont stages being secreted in the lumen of the parasitophorous vacuole [3], estimated by proteomic studies to be one of the most abundant protein expressed at the schizont stage [4]. Upon release of merozoites from mature schizonts, P126 is processed into fragments of 50 and 73 kD, the latter composed of two peptides of 47 and 18 kD linked by disulfide bridges. These fragments are released into the bloodstream [5], the complex of 47 and 18 kDa peptides is also known to be adsorbed onto the surface of free merozoites [6,7]. The P126 protein contains cysteine protease domains [8], suggesting that it may have an essential function in merozoite release [9] and reinvasion. Monoclonal and polyclonal antibodies against P126 are able to inhibit parasite growth *in vitro*, and a major parasite-inhibitory epitope has been recently mapped to its 47 kDa N-terminal extremity (octamer repeats domain – OR domain) [10,11]. Moreover, immunization of *Saimiri *and *Aotus *monkeys with different fractions of P126, including the Nt47 domain [1,12-15], induced antibodies that protected monkeys against challenge infection. Immuno-epidemiological studies performed in holoendemic (Uganda) and mesoendemic (Brazil) areas have displayed a positive association between naturally acquired cytophylic IgG antibody response to the OR domain and an increased protective immunity in adults [16,17] and children [16,18], suggesting that cellular effector mechanisms, such as antibody-dependent cellular inhibition targeting the P126 protein, might play a primary role in protection against malaria. However, it was observed that malaria-infected people with different degrees of exposure, as naïve tourists experiencing their first infection or chronically exposed individuals from areas exhibiting a different level of endemicity (Senegal and Brazil), developed anti-OR antibodies with similar prevalence (77%) [17]. The lack of response in the remaining 23% of the patients could be due to a genetic restriction of the immune response to the OR domain. In fact, in previous work there were significant associations between anti-OR response and the presence of HLA-DR4, and the absence of anti-OR response with the presence of HLA-DR15 [19]. Therefore, genetic restriction alone cannot explain these findings since not all HLA-DR15 individuals were non-responders as well as not all HLA-DR4 individuals were responders. Genetic diversity of *P. falciparum *antigen repetitive regions is thought to contribute to immune invasion and also appear to restrict the effectiveness of subunit vaccines. In fact, although the P126 structure has been proven to be remarkably conserved among *P. falciparum *isolates, the P126 gene is a member of a multigene family [20]. Most polymorphism has been described in exon II which corresponds to the amino terminal region (47 kDa peptide) of the protein that encodes two repeat domains: the octamer repeats (Nt47 = OR domain) and the serine repeats (SR domain) characterized by a stretch of serine residues. The P126 polymorphisms basically comprise the events of deletions/insertions in these two repetitive domains of the protein, but nucleotide replacements, synonymous or not, were also described [21-24]. The OR region predominately consists of six octamer units (OR-II), but variation in the sequence and number of repeat units (5 octamer units – OR-I) may be seen in different alleles [24]. The occurrence of polymorphism in the OR domain, which encodes B- and T-epitopes [25] involved in the induction of the protective immunity, may provide a mechanism for parasites to evade immune response induced by either natural exposition to infections or vaccine. Recently, analyses of *P. falciparum *isolates (n = 52) from three areas of the Brazilian Amazon have identified the occurrence of the two P126 OR fragments, the most frequent constituting six octamer repeats [26]. The study of P126 OR domain polymorphism influence on the naturally acquired specific antibody responses in individuals living in malaria-endemic areas may provide valuable insights for vaccine development against this high priority disease. In this context, the goal of this study was to extend the knowledge of P126 OR domain genetic polymorphism in Brazilian *P. falciparum *isolates (by PCR-SSCP and sequencing) and evaluate its impact on the natural OR-antibody responses in malaria-exposed Brazilian Amazon individuals.

## Methods

### Study sites

The study was carried out in two villages, Candeias do Jamari (8°48'35"S; 63°41'44"W) in Rondonia state and Peixoto de Azevedo (10°13'23"S; 54°58'47"W) in Mato Grosso state, both located in the south-western part of the Amazon region. This region became the target of a large influx of people from other Brazilian regions during the 70s and 80s and malaria is hypoendemic to mesoendemic and is present throughout the year with seasonal fluctuations. The economic activities in both villages were subsistence farming, but gold-mining have become an attractive trade to Peixoto de Azevedo village. The population of Candeias do Jamari is composed of natives (30%) and migrants (70%) inhabiting this area for variable times since the 1970s. The first sample set were collected in 1993 (Candeias do Jamari-1993) and the second sample set was obtained nine years later, in 2002 (Candeias do Jamari-2002). The average annual parasite incidence (API), in Candeias do Jamari, was 549 in 1993 and 173 in 2002. For reference, the Brazilian Ministry of Health considers high risk areas those with API ≥ 50. The population of Peixoto de Azevedo is consisted mainly of gold miners and they frequently come from other gold prospecting places inside the Amazon. The samples were collected in 1995 in Peixoto the Azevedo (PA group) when the API was 292. Malaria transmission in both villages occurs throughout the year, with seasonal fluctuations with an increase of incidence at the beginning and at the end of dry season when all samples were collected.

### Patients and isolates

Written informed consent was obtained from all donors and venous blood samples were taken from *P. falciparum *infected individuals: 96 from Peixoto de Azevedo in 1995, 103 from Candeias do Jamari in 1993 (Candeias do Jamari-1993 group) and 78 Candeias do Jamari in 2002 (Candeias do Jamari-2002 group). OR fragments polymorphism in samples from three cohorts (CJ 1993, CJ 2002 and PA) were used in order to evaluate whether the OR fragment polymorphism in Brazil was present and appeared to be temporally stable in the same area (1993 and 2002) or if it was spread throughout different areas (Candeias do Jamari and Peixoto de Azevedo). All *P. falciparum *malaria patients who were enrolled in this study complied with the following criteria: 1) they presented symptoms; 2) they had unique *P. falciparum *thick blood smears; 3) they neither used chemoprophylaxis nor took antimalarial drugs (self-treatment); 4) they were all over 15 years old; 5) no females were pregnant or breast feeding and; 6) blood collection was performed the day of diagnosis before malaria treatment. The patients sought health care at Brazilian health services 3.6 ± 1.8 days after onset of symptoms. After blood sample collection, the patients were immediately treated according to Brazilian Ministry of Health standards for malaria therapy with quinine plus doxycycline and primaquine.

Thin and thick blood smears were examined for identification of malaria parasite by two expert malaria microscopists from Brazilian Malaria Health Services and from the Laboratory of Malaria Research (Fiocruz) which is a reference in malaria diagnosis from Brazilian Ministry of Health. Thick blood smears from all subjects were stained with Giemsa, and a total of 200 microscopic fields were examined under 1000-fold magnification. Thin blood smears of positive samples were examined for species identification. Only patients infected with *P. falciparum *in the thick and thin blood smears were enrolled in our study. The *P. falciparum *parasite density was determined by counting parasites in a predetermined number of white blood cells in thick blood films, and the number of blood parasites per milliliter was calculated [27]. The fresh blood samples were washed three times with 0.15 M phosphate-buffered saline and pellet containing packed red blood cells was mixed with equal volume of cryopreservation solution (0.9% NaCl/4.2% sorbitol/20% glycerol) and frozen in liquid nitrogen (N_2_).

### DNA extraction

The isolation of DNA from blood was carried out using the method previously described [28]. One μL of red blood cells was lysed by saponin lysis buffer (15%) and then centrifuged at 7,000 × *g *for 10 min. The pellets were mixed with 300 μL of NET buffer (150 mM NaCl, 10 mM EDTA, and 50 mM TRIS, pH 7.0) containing 0.1% (w/v) Sarcosyl and 200 μg proteinase K and were kept for 3 h at 42°C. The sample was then extracted once with phenol/clorophorm/isoamyl alcohol (25/24/1 w/v) and then once with clorophorm/isoamyl alcohol (24/1 w/v). The DNA was precipitated with cold ethanol and reconstituted in 50 μL of TE buffer (10 mM TRIS-HCl and 1 mM EDTA, pH 8.0).

### Amplification of the P126 gene fragment and electrophoresis

The repetitive region of the gene encoding the P126 antigen was amplified in a Nested PCR method using the first set of primers *p126A *(5'-AAT GAA GTC ATA TAT TTC CTT G-3') and *p126B *(5'-CAA TGT TGT TCT TAA TTC GAT A-3') and the second set of primers *p126C *(5'-GTG TTA TAT TTA ACA AAA ATG-3') and *p126D *(5'-CTT ACA GGA TTG CTT GGT TCG-3') [29]. One μL of DNA was amplified in a 50 μL reaction volume containing 10 nmol of each deoxynucleotide triphosphate (dNTP, Promega, Madison, WI), 100 pmoles of each primer (Invitrogen, USA), 2.5 U of AmpliTaq^® ^DNA Polymerase (Applied Biosystems, Foster City, CA) and 5 μL of 10× buffer (2.0 mM MgCl_2_, Promega, Madison, WI). Both single and nested PCR reactions were carried out using a GeneAmp^® ^PCR System 9700 (Applied Biosystem, Foster City, CA) for thirty cycles (1 min. at 94°C, 2 min. at 47°C, 3 min. at 72°C). Ten μL of PCR reaction were loaded onto a 2.5% agarose gel (Sigma, Missouri, USA) in 1× TAE buffer (0.04 M TRIS-acetate, 1 mM EDTA) in the presence of ethidium bromide (0.5 μg/mL).

### Single-strand conformational polymorphism (SSCP)

In order to assess sequence microheterogeneity within fragments defined as the same genotype by PCR, each amplified fragment was analysed by SSCP. The PCR sample was previously concentrated with 0,3 M of sodium acetate pH 5.2, recovered in 20 μL of distilled water and loaded onto a 2% Nusieve GTG agarose gel (FCM Bioproduct, USA) in 1 × TAE buffer (0.04 M TRIS-acetate, 1 mM EDTA). The amplified fragments were individually gel-purified using Wizard PCR preps (Promega, Madison, WI) as recommended by suppliers and resuspended in 40 μL of distilled water. PCR fragments were digested to completion with *RsaI *(Promega, Madison, WI) and then extracted with phenol-chloroform and precipitated with ethanol. The samples were denatured prior to analysis by adding one μL of 0.5 M NaOH/10 mMEDTA and then electrophoresed for 3 h at 10 V/cm using 0.5 × TBE buffer (pH8.4). SSCP patterns were visualized by silver staining as described elsewhere [30].

### DNA Sequencing

Uncloned amplified fragments were sequenced using the di-deoxy chain termination procedure with *Taq *polymerase and fluorescently labeled (Big Dye Terminator Kit version 3.1, Applied Biosystems, Foster City, CA) with the primers *p126C *5' GTG TTA TAT TTA ACA AAA ATG 3' (forward) and *p126D *5' CTT ACA GGA TTG CTT GGT TCG-3' (reverse). Nucleotide sequences were obtained by the automated method (DNA sequencer model 3100 Genetic Analyzer; Applied Biosystems, Foster City, CA) and the sequences assembled by using SeqMan II, included in the DNASTAR software package (Madison, Wis.). The OR domain sequences reported for field isolates have been submitted to EMBL/GenBank Data libraries under accession number EU360815 and EU360816.

The graphical representations of the predicted antigenic determinants on the OR domains were generated using the program DNASTAR software package (Madison, Wis.) and the antigenic indexes were calculated using the method described by Jameson & Wolf [31].

### Nt47 peptide

The Nt47 synthetic peptide corresponding to the six octamer repeats (OR-II) of the P126 amino terminal region [32,33] was prepared using a solid-phase method [34] as previously described [35]. The amino acid sequence of the Nt47 is Thr Gly Glu Ser Gln Thr Gly Asn – Thr Gly Gly Gly Ala Gly Asn – Thr Gly Gly Gly Gln Ala Gly Asn – Thr Val Gly Asn Gln Ala Gly Ser – Thr Gly Gly Ser Pro Gln Gly Ser – Thr Gly Ala Ser Gln Pro Gly Ser – Cys. The OR-II domain was selected instead of OR-I (five octamer repeats) because in the ELISA competition assay using human sera from endemic regions that recognize Nt-t47 react with peptides containing at least four repeats [17]. In addition, the OR-II domain is predominantly present in *P. falciparum *parasite isolates from different malaria endemic regions [24].

### Enzyme-linked immunosorbent assay (ELISA) for Nt47 (OR-II domain)

The ELISA was performed as previously described [17,19]. Briefly, microtiter 96-well plates (Maxisorp, NUNC, Denmark) were coated overnight at 4°C with 2 μg/mL of Nt47 peptide in 0.05 M NaHCO3 pH 9.6. After blocking with 4% bovine serum albumin (BSA)/0.05%Tween 20 in PBS (1 hr at 37°C) and washing with Milli-Q water – 0.05% Tween 20, human sera diluted in 1% BSA/0.05% Tween 20/PBS (1:100) were added to the plates. After incubation (1 hr at 37°C), the plates were washed and appropriate peroxidase-conjugated anti-human IgG or IgM (Sigma, Missouri, USA) in 1% BSA/0.05% Tween 20/PBS was added and incubated for 1 hr at 37°C. In order to detect specific IgG subclasses, plates were incubated for 2 hr at 37°C with murine MAb labeled with peroxidase specific for human IgG1 (clone 4E3), IgG2 (clone 31-7-4), IgG3 (clone HP 6050) and IgG4 (clone HP 6025) subclasses (Southern Biotech, Alabama, USA). Bound antibodies were detected with the substrate orthophenylediamine (Merck, Rio de Janeiro, Brazil) and H_2_O_2 _in citrate-phosphate buffer pH 5.0. Plates were read at 492 nm in a spectrophotometer (Spectramax 250, Molecular Devices). Each sample was tested in duplicate. Sera from 25 control individuals living in Rio de Janeiro and never exposed to malaria transmission, were used to establish the normal range for the assay. The cutoff value was determined as the mean optical density (OD) 3 standard deviations of the Rio controls (cutoff values: IgG = 0.135; IgM = 0.168; IgG1 = 0.138; IgG2 = 0.159; IgG3 = 0.142; IgG4 = 0. 172). To standardize the OD data obtained in different experiments and to evaluate the specific antibody levels, an OD index was calculated for each immunoglobulin determination as the ratio between the absorbance of each tested samples and the cutoff values. Sample with OD index > 1.0 was considered positive.

### Statistical analysis

The data were stored in the Fox-plus^® ^(Borland International, Inc. Perrysburg, OH) data bank software. Statistica (Microsoft, Inc. Redmond, WA) and Epi-Info 6 (Centers for Disease Control and Prevention, Atlanta, GA) statistical software programs were used for data analysis. The Student's *t*-test was used to analyse the differences in mean values, the chi-square test was used to analyse the difference in prevalence of positive responses and of allelic frequencies, and the Spearman rank coefficient test used to analyse the correlations between variables.

## Results

### Characteristics of the studied groups

Throughout the study period, 277 *P. falciparum *samples were collected from malaria patients from Candeias do Jamari-1993 (n = 103), Candeias do Jamari-2002 (n = 78) and Peixoto de Azevedo (n = 96). The average age was similar among individuals from Candeias do Jamari-1993 (30 ± 12 years old), Candeias do Jamari-2002 (33 ± 14 years old) and Peixoto de Azevedo (29 ± 10 years old). The time of residence of individuals in the endemic regions were 13 ± 10 years in Candeias do Jamari-1993, 21 ± 11 years in Candeias do Jamari-2002 and 4 ± 3 years in Peixoto de Azevedo, the Candeias do Jamari-1993 and Candeias do Jamari-2002 groups residing on average longer in a malaria endemic area than that in Peixoto de Azevedo (Student's *t*-test; *P *< 0.0001, for both). As expected, the Candeias do Jamari-2002 group had a higher mean time of residence in the malaria endemic area than the Candeias do Jamari-1993 group (Student's *t-*test; *P *< 0.0001). The mean parasitaemia at the time of blood collection were 9,642 ± 10,301, 2,150 ± 654 and 9,288 ± 9,058 parasites/μL in the Candeias do Jamari-1993, Candeias do Jamari-2002 and Peixoto de Azevedo groups, respectively. The Candeias do Jamari-2002 group demonstrated a lower parasitaemia than the Candeias do Jamari-1993 and Peixoto de Azevedo groups (Student's *t-*test; *P *< 0.0001, for both). The great majority of the individuals claimed to have experienced malaria episodes previously. Only 5% (n = 9) of the individuals from Candeias do Jamari-1993 and 2002 and 9% (n = 9) of the individuals from Peixoto de Azevedo denied prior malaria infection.

### Genetic polymorphism of P126

Only two types of OR fragments were identified in the studied areas by PCR-SSCP analysis, one of 175 bp (OR-I) and the other of 199 bp (OR-II). The frequencies of OR-I and OR-II fragments found in *P. falciparum *wild isolates were, respectively, 7.4% and 92.6% in Candeias do Jamari-1993 and 9.8% and 90.2% in Candeias do Jamari-2002. Predominance of the OR-II fragment was evident in the *P. falciparum *samples from Candeias do Jamari both 1993 and 2002. Curiously, a different pattern appeared in relation to OR fragment frequencies in Peixoto de Azevedo where OR-I fragment frequency (40.5%) was slightly lower than the OR-II fragment (59.5%). In Peixoto de Azevedo there was a higher frequency for the OR-I fragment when compared to Candeias do Jamari-1993 and Candeias do Jamari-2002 (Chi-square test; *P *< 0.0001, for both Candeias do Jamari groups) while the frequency of the OR-II fragment was lower (Chi-square test; *P *< 0.0001, for both Candeias do Jamari groups).

Table 1 presents the frequency of single and mixed infections according to the groups. As can be seen, the complexity of the infection in Candeias do Jamari was similar in the 1993 and 2002 surveys, and there was a *P. falciparum *mixed infection with OR-I and OR-II fragments in 4.9% (Candeias do Jamari-1993) and in 5.1% (Candeias do Jamari -2003). It is interesting to note that in Peixoto de Azevedo, there was a higher frequency of mixed infection (20.8%) than in Candeias do Jamari *P. falciparum *isolates (Chi-square test; *P *= 0.001 PA *versus *CJ-1993; *P *= 0.0034 PA *versus *CJ-2002). No relationship was established between infection patterns and age, sex or time of residence in endemic areas of Candeias do Jamari-1993, Candeias do Jamari-2002 and Peixoto de Azevedo.

**Table 1:** Infection patterns of the *Plasmodium falciparum *isolates from Candeias do Jamari and Peixoto de Azevedo analysed by Nested PCR.

**Complexity** ** infection**	**OR** **fragments** (bp)	**Candeias do Jamari (CJ)**		**Peixoto de Azevedo** ** (PA)**
		**1993**	**2003**	
				
		n (%)		n (%)
Single infections^1^				
	I (175)	3 (3%)	4 (5%)	27 (28%)^a, b^
	II (199)	95 (92%)	70 (90%)	49 (51%)^c, d^
Mixed infection^2^				
	I + II (175/199)	5 (5%)	4 (5%)	20 (21%)^e, f^

**Total**		103 (100%)	78 (100%)	96 (100%)

^1^Isolates with one fragment; ^2^Isolates with two fragments; bp: base pair; n: number of isolates (the percentage of isolates is given in parentheses); a: *P *< 0.0001, PA > CJ-1993; b: *P *< 0.0001, PA > CJ-2002; c: *P *< 0.0001, PA < CJ-1993; d: *P *< 0.0001, PA < CJ-2002; e: *P *= 0.001, PA > CJ-1993; f: *P *= 0.003, PA > CJ-2002. The chi-square test was used to analyse the difference in allelic frequencies.

### DNA sequencing of the OR domain (Nt47)

**Figure 1:**
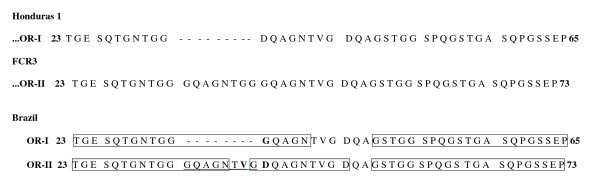
**Alignment of the predicted amino acid sequences of P126 OR region from Candeias do Jamari and Peixoto de Azevedo *P. falciparum *isolates** (GenBank accession number EU360815 and EU360816). The OR fragments OR-I and OR-II from Brazil were compared one each other and to FCR3 and Honduras1 OR described sequences reported by Morimatsu and others [22] and Liu and others [35]. The motif insertion is underlined, the lake of corresponding residues is indicated by dash, different residues are indicated in boldface letters and two (OR-I) or three (OR-II) antigenic determinants are in box.

**Figure 2:**
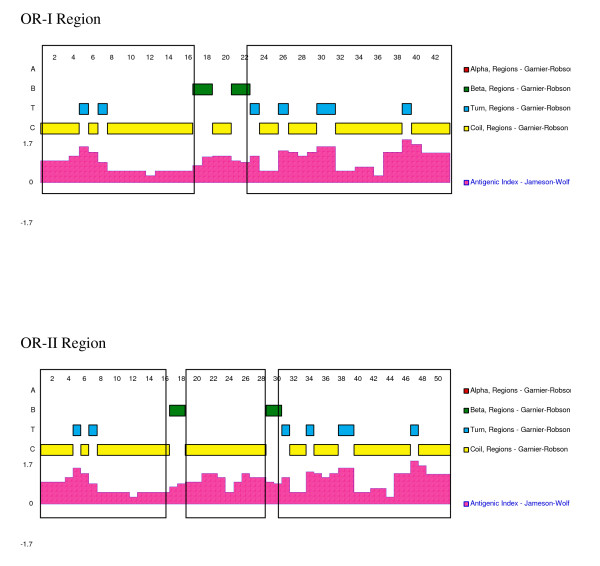
**Comparison of the predictive regions of the OR domain**. The two (OR-I) or three (OR-II) antigenic determinants are in box.

Comparing the DNA sequencing of OR-I and OR-II fragments of wild *P. falciparum *isolates from Candeias do Jamari-1993, Candeias do Jamari-2002 and Peixoto de Azevedo, a high conservation among predicted amino acid sequences of the P126 N-terminal extremity was evident. As shown in Figure 1, OR-I and OR-II fragments of Brazilian wild parasite isolates differ from each other by the insertion of the octamer unit (GQAGNTVG) between amino acid residues 33 and 34. The OR-I Brazilian fragment corresponded to the Honduras 1-type, differing only by one non-synonymous amino acid replacement at the 42nd (D→G) position, and the OR-II Brazilian fragment corresponded to the FCR3-type, differing by the two non-synonymous amino acid replacement at the 40th (G→V) and 42nd (G→D) positions. The computer-predicted an antigenic index of the OR-I and OR-II domains from Brazilian isolates, demonstrating that in spite of the OR-I and OR-II domains presenting a similar antigenic-index scores (0.86 and 0.87, respectively), OR-II contains 3 antigenic sites whereas OR-I contains 2 (Figure 2).

### Antibody responses to the OR domain regardless of the infection patterns

The Nt47 synthetic peptide, which is based on the six octamer repeat sequence located at the N-terminal extremity of P126 (OR-II), was used in ELISA to detect specific IgM, IgG and IgG subclasses. Most of the individuals were tested for humoral immune responses against the OR-II domain.

There was a predominance of OR domain antibody responders, regardless of the class (IgG or IgM), in the Candeias do Jamari-2002 group (92%) when compared with those observed in Candeias do Jamari-1993 (78%, *P *= 0.01 by chi-square test) or Peixoto de Azevedo (80%, *P *= 0.02 by chi-square test) (Total – Table 2). This predominance was not associated with either the time of residence in a malaria endemic area, sex or age. However, in the Candeias do Jamari-1993 and Peixoto de Azevedo groups, the parasitaemia was significantly lower in OR antibody responders (mean = 7,857 ± 8,688 parasites/μL; 8,356 ± 8,070 parasites/μL, respectively) compared to non-responders (mean = 16,504 ± 13,257 parasites/μL, *P *= 0.04; 14,284 ± 12,081 parasites/μL, *P *= 0.01, respectively, by Student's *t*-test). As shown in Table 3, the prevalence of OR IgG responders in Candeias do Jamari-1993 (70%) and Candeias do Jamari-2002 (74%) was not statistically different than that observed in the Peixoto de Azevedo group (79%). However, the IgG antibody levels (OD index) were higher in Candeias do Jamari-2002 (8.6 ± 8.3) than in the Candeias do Jamari-1993 (4.9 ± 3.0) or Peixoto de Azevedo (4.5 ± 2.8) groups (Student's t-test; *P *< 0.05, for both – Table 3). In relation to anti-OR IgM antibodies the prevalence and the levels were higher in Candeias do Jamari-2002 (81% and 4.9 ± 4.8) than that observed in either Candeias do Jamari-1993 (63% and 3.4 ± 2.0) or Peixoto de Azevedo (54% and 2.3 ± 1.5) (Chi-square test and Student's *t*-test; *P *< 0.05, for all analyses – Table 3). No association was derived with respect to age, sex, parasite densities or time of residence in a malaria endemic area and the prevalence or levels of OR IgG and IgM antibody responses in any of three groups studied (Chi-square test, Student's *t*-test and Spearman rank coefficient test; *P *> 0.05, for all).

**Table 2:** Prevalence of anti-OR antibody responses in Candeias do Jamari and Peixoto de Azevedo groups according to the infection patterns.

Infection patterns	Candeias do Jamari (CJ)		Peixoto de Azevedo (PA)
	1993	2002	

Single^1^			
*OR-I*	3/3	3/4	21/25 (84)
*OR-II*	52/69 (75)	65/70 (93)^a, b^	36/46 (78)
Mixed^2^	4/4	4/4	16/20 (80)

**TOTAL**	**59/76 (78)**	**72/78 (92)**^c, d^	**73/91 (80)**

^1^Isolates with one fragment; ^2^Isolates with two fragments. The data represent the number of responders/number of tested individuals (the percentage of responders is given in parentheses). a: *P *= 0.005, CJ-2002 OR-II > CJ-1993 OR-II; b: *P *= 0.04, CJ-2002 > PA OR-II; c: *P *= 0.01, CJ- 2002 > CJ-1993; d: *P *= 0.02, CJ- 2002 > PA. The Chi-square test was used to analyse the difference in prevalence of positive responses.

**Table 3:** Prevalence and levels of antibodies to the OR domain of the *P. falciparum *P126 protein in Candeias do Jamari and Peixoto de Azevedo groups regardless of the infection patterns.

	**PA (*a*)**	**CJ-1993 (*b*)**	**CJ-2002 (*c*)**	***P *values**
**IgG**	71/90 (79%)	53/76 (70%)	58/78 (74%)	n.s.
	4.5 ± 2.8	4.9 ± 3.0	8.6 ± 8.3	*a *× *c*: 0.0006; *b *× *c*: 0.002
**IgM**	49/90 (54%)	43/68 (63%)	63/78 (81%)	*a *× *c*: 0.0006; *b *× *c*: 0.02
	2.3 ± 1.5	3.4 ± 2.0	4.9 ± 4.8	*a *× *c*: 0.0002; *b *× *c*: 0.04
**IgG1**	45/70 (64%)	43/49 (87%)	14/58 (24%)	*a *× *b*: 0.007; *a *× *c*: < 0.0001; *b *× *c*: < 0.0001
	2.3 ± 2.0	3.2 ± 2.0	1.9 ± 0.7	*b *× *c*: 0.001
**IgG2**	5/70 (7%)	7/49 (14%)	21/58 (36%)	*a *× *c*: 0.0001; *b *× *c*: 0.01
	1.3 ± 0.4	2.8 ± 2.8	1.3 ± 0.4	n.s.
**IgG3**	68/70 (97%)	47/49 (96%)	35/58 (60%)	*a *× *c*: < 0.0001; *b *× *c*: < 0.0001
	8.1 ± 6.4	10.2 ± 9.7	7.9 ± 6.1	n.s.
**IgG4**	30/70 (43%)	23/49 (47%)	24/58 (41%)	n.s.
	1.3 ± 0.2	1.6 ± 0.7	2.5 ± 2.2	n.s.

The data represent the number of responders/number of tested individuals (the percentage of responders is given in parentheses) and the antibody levels expressed as mean of OD index ± SD. The Student's *t*-test was used to analyse the differences in mean values, the Chi-square test was used to analyse the difference in prevalence of positive responses.

For the majority of individuals with a detectable IgG-OR antibody, subclasses of IgG were determined in Candeias do Jamari-1993 (49 of 53, 92%), Candeias do Jamari-2002 (58 of 58, 100%) and Peixoto de Azevedo (70 of 71, 98%) groups. As shown in Table 3, the IgG subclass profiles were different in the Candeias do Jamari-2002 (IgG3 = 60%; IgG4 = 41%; IgG2 = 36%; IgG1 = 24%) group compared to those observed in Candeias do Jamari-1993 (IgG3 = 96%; IgG1 = 87%; IgG4 = 47%; IgG2 = 14%) or with Peixoto de Azevedo (IgG3 = 97%; IgG1 = 64%; IgG4 = 43%; IgG2 = 7%). There was a predominance of IgG1 and IgG3 OR-specific antibodies (cytophilic) over IgG4 and IgG2 (noncytophilic) in the Candeias do Jamari-1993 and Peixoto de Azevedo groups (Chi-square test; *P *< 0.001, for all analyses – Table 3), whereas in Candeias do Jamari-2002 there was a predominance of IgG3 OR-specific antibodies (Chi-square test; IgG3 > IgG1 and IgG2, *P *< 0.01, for both analyses) the prevalence of IgG1 and IgG3 OR-specific antibodies were significantly lower when compared with Candeias do Jamari-1993 and Peixoto de Azevedo groups (Chi-square test; *P *< 0.05, for all analyses – Table 3). However, in Candeias do Jamari from 1993 to 2003 the antibody levels were similar except for IgG1 that was significantly lower in 2002 (Student's *t*-test; *P *< 0.001 – Table 3). The levels of OR IgG3 antibodies in each group significantly exceeded those of other subclasses (Student's *t*-test; *P *< 0.001, for all analyses – Table 3). No association was derived with respect to age, sex, parasite densities or time of residence in a malaria endemic area and the prevalence or levels of OR IgG antibody subclass in any of three groups studied (Chi-square test, Student's *t*-test and Spearman rank coefficient test; *P *> 0.05, for all).

### Antibody responses to the OR domain according to the infection patterns

Due to the small number of individuals with single (only OR-I) or mixed (OR-I and OR-II simultaneously) *P. falciparum *infections in both Candeias do Jamari-1993 and Candeias do Jamari-2002, the statistical analysis of antibody responses to the OR domain according to the infection patterns was performed only in the Peixoto de Azevedo. In this group the prevalence and/or the levels of OR-specific antibodies regardless of class and subclass – (Table 2 and Table 4) were broadly similar among the groups infected with either single or mixed *P. falciparum *isolates (Chi-square test and Student's *t*-test; *P *> 0.05, for all analyses).

**Table 4:** Prevalence and levels of antibodies to the OR domain of the *P. falciparum *P126 protein in Candeias do Jamari and Peixoto de Azevedo groups according to the infection patterns.

**Infection**	**IgG**	**IgM**	**IgG1**	**IgG2**	**IgG3**	**IgG4**
	Candeias do Jamari (CJ)					

**OR-II**						
**CJ-1993**	47/69 (68) 4.9 ± 3.0^d^	39/63 (62) 3.4 ± 2.1	39/44^a^ (88) 3.2 ± 2.1^e^	6/44^b^ (13) 3.1 ± 2.9	42/44^c^ (95) 10.6 ± 10.0	19/44 (43) 1.6 ± 0.7^f^
**CJ-2002**	55/70 (78) 8.4 ± 8.2^j^	57/70^g ^(81) 4.8 ± 4.8	13/55^h ^(23) 1.9 ± 0.7	19/55^i ^ (34) 1.3 ± 0.4	35/55 (60) 7.5 ± 6.1	23/55 (42) 2.6 ± 2.2^k^

	Peixoto de Azevedo (PA)					

**OR-I**	19/25 (76) 4.9 ± 2.9	13/25 (52) 1.7 ± 0.9	13/18^l ^(72) 2.0 ± 1.8	0/18^m^ -	18/18^n ^(100) 9.8 ± 6.8	9/18 (50) 1.4 ± 0.2
**OR-II**	36/46 (78) 4.3 ± 2.7	27/46 (59) 2.7 ± 1.7	21/36^o ^(58) 2.1 ± 1.7	5/36^p ^(14) 1.3 ± 0.4	34/36^q ^(94) 7.6 ± 6.1	16/36 (44) 1.3 ± 0.1
**Mixed**	16/19 (84) 4.5 ± 3.1	9/19 (47) 2.0 ± 1.4	11/16^r^(69) 2.9 ± 2.7	0/16^s^ -	16/16 (100)^t^ 7.5 ± 6.4	5/16 (31) 1.3 ± 0.1

The data represent the number of responders/total of each fragment tested (the percentage of responders is given in parentheses) and the antibody levels expressed as mean of OD index ± SD. *P *< 0.05. **a: **CJ-1993- IgG1 > IgG2, IgG1 > IgG4; CJ-1993 IgG1 > CJ-2002 IgG1; **b**: CJ-1993- IgG2 < IgG3, IgG2 < IgG4; CJ-1993 IgG2 < CJ-2002 IgG2; **c**: CJ-1993- IgG3 > IgG4; CJ-1993 IgG3 > CJ-2002 IgG3; **d: **CJ-1993 IgG < CJ-2002 IgG; **e: **CJ-1993 IgG1 > CJ-2002 IgG1; **f: **CJ-1993 IgG4 < CJ-2002 IgG4; **g: **CJ-2002 IgM > CJ-1993 IgM, CJ-2002 IgM > PA IgM; **h: **CJ-2002 IgG1 < IgG3; **i**: CJ-2002 IgG2 < IgG3; CJ-2002 IgG2 > PA OR-II IgG2; **j: **CJ-2002 IgG > PA OR-II IgG; **k: **CJ-2002 IgG4 > PA OR-II IgG4 **l**: PA OR-I IgG1 > IgG2; PA OR-I IgG1 < IgG3; **m**: PA OR-I IgG2 < IgG3; PA OR-I IgG2 < IgG4; **n**: PA OR-I IgG3 > IgG4; **o**: PA OR-II IgG1 > IgG2; PA OR-II IgG1 < IgG3; **p**: PA OR-II IgG2 < IgG3; PA OR-II IgG2 < IgG4; **q: **PA OR-II IgG3 > IgG4; CJ-2002 IgG3 < PA OR-II IgG3; **r: **PA mixed IgG1 > IgG2; **s: **PA mixed IgG2 > IgG3; **t**: PA mixed IgG3 > IgG4. The Student's *t*-test was used to analyse the differences in mean values, the Chi-square test was used to analyse the difference in prevalence of positive responses.

Comparing the prevalence of OR antibody responders in the three groups of individuals infected with the *P. falciparum *OR-II fragment, the prevalence of OR IgM antibody responders was higher in Candeias do Jamari-2002 (81%) than in the Candeias do Jamari-1993 (62%, *P *= 0.01) or Peixoto de Azevedo (59%, *P *= 0.01) groups (Table 4). However, the OR IgM antibody levels in the Candeias do Jamari-2002 group were only slightly higher than in the Candeias do Jamari-1993 or Peixoto de Azevedo groups (Student's *t*-test; *P *> 0.05 – for all analyses – Table 4).

Comparing the profiles of OR IgG subclasses among the three groups of individuals infected with the *P. falciparum *OR-II fragment (Table 4), there was a predominance of IgG1 and IgG3 OR-specific antibodies (cytophilic) over IgG4 and IgG2 (noncytophilic) in the Candeias do Jamari-1993 and Peixoto de Azevedo groups (Chi-square test; *P *< 0.001, for all analyses), whereas in Candeias do Jamari-2002 there was a predominance of IgG3 OR-specific antibodies (Chi-square test; IgG3 > IgG1 and IgG2, *P *< 0.01, for both analyses). The prevalence of IgG1 and IgG3 OR-specific antibodies was significantly lower in Candeias do Jamari-2002 when compared with Candeias do Jamari-1993 and Peixoto de Azevedo groups (Chi-square test; *P *< 0.05, for all analyses). However, in Candeias do Jamari from 1993 to 2003 the antibody levels were similar except for IgG1 that was significantly lower in 2002 (Student's *t*-test; *P *< 0.001 – Table 3). The levels of OR IgG3 antibodies in each group significantly exceeded those of other subclasses (Student's *t*-test; *P *< 0.0008, for all analyses). No association was derived with respect to age, sex, parasite densities or time of residence in a malaria endemic area and the prevalence or levels of OR IgG antibody subclass in any of three groups studied (Chi-square test, Student's *t*-test and Spearman rank coefficient test; *P *> 0.05, for all).

## Discussion

The aims of this study were to investigate the polymorphism of the P126 N-terminal region octamer repeat domain (OR = Nt47 domain) in *P. falciparum *isolates from two important Brazilian malaria endemic areas (Candeias do Jamari in 1993 and 2002 and Peixoto de Azevedo in 1995) and assess the impact of OR fragment polymorphism on specific, naturally acquired antibodies in the studied populations.

In Africa, Asia and South America, including Brazil [24,26,36], only one single OR fragment type (OR-I or OR-II) appeared in *P. falciparum *malaria infected individuals while in our study, mixed infection (both OR-I and ORII) was detected for the first time in inhabitants of both villages with high frequency in Peixoto de Azevedo. Also noteworthy are the differences in the OR fragment frequencies among *P. falciparum *isolates from Peixoto de Azevedo when compared with the isolates from other endemic areas. In Peixoto de Azevedo the OR-I fragment frequency (40.5%) was only slightly lower than OR-II (59.5%). The high mixed infection and single OR-I fragment prevalence in Peixoto de Azevedo in comparison with isolates from the Candeias do Jamari group as well as groups from a myriad of other geographical areas, is probably due to: i) an immunological status of this population, since studied individuals from this village, mainly migrants from malaria-free areas, have been inhabiting a malaria endemic area for an average of four years, ii) a relatively recent introduction of the variant into this population as a consequence of the gold miners' nomadic behaviour within the Amazon, facilitating new variant spread throughout the region and iii) the differences in the biological development of this variant in man and/or its invertebrate host. Although in a Brazilian endemic area [26] only single infection had previously been detected, this study's relatively small sample size may account for an underestimation of mixed infection scope.

It is well recognized that the complexity of infections may vary considerably in different epidemiological scenarios according to age and immune status of the individuals [37,38]. In the study areas, remarkably no relationship was established between infection patterns and age, sex or time of residence in a malaria endemic area.

The sequences herein analysed, further confirm a limited degree of genetic polymorphism in the P126 OR domain, namely variation in the number of octamer repeats (OR-I five or OR-II six) and non-synonymous substitutions in repeat units [24,26,36]. In addition, there was a remarkable similarity between the P126 OR domain of *P. falcipar*um from Candeias do Jamari in 1993 and 2002, a difference of 9 years, suggesting that this portion of the P126 gene was both genetically homogeneous and temporally stable, and despite differing from the Candeias do Jamari groups in terms of frequencies and infection complexity, the Peixoto de Azevedo group shared an identical degree of polymorphism in the P126 OR domain. These results are similar to a study in the same endemic areas with the MSP2 protein [39] that revealed genetic similarity between Brazilian isolates from the same areas. Considering the limited polymorphism of P126 also reported in other studies in wild and laboratory isolates from Africa, Asia and South America [21-24,36], the finding of similar sequences in isolates from the same endemic area or different geographical areas may be expected. The high degree of conservation of diverse alleles moreover supports the hypothesis of a biologically important role for P126 in parasite development and/or survival.

Based on the fact that B and T-cell epitopes are located in the OR domain [25], variation in the number of octamer repeats and non-synonymous substitutions in repeat units could result in major changes in the affinity of the antigen-receptor interactions (B-cell receptor, T-cell receptor and MHC molecules) by altering epitope chemical properties and structure [40], hence theoretically modulating the host immune response, affording parasite evasion. The computer-predicted antigenic index of the OR-I and OR-II domains from Brazilian isolates demonstrated that although the OR-I and OR-II domains present similar antigenic-index scores (0.86 and 0.87, respectively), OR-II contains three antigenic sites whereas OR-I contains only two (Figure 2). Indeed, the prevalence of antibody responses to the P126 OR domain was very high in all studied groups regardless of the infection patterns (OR-I, OR-II or mixed). The high prevalence of antibodies to the OR domain in all three studied groups illustrates that the P126 OR domain is highly immunogenic in man, which is in agreement with data from holoendemic [16,18,25,41] and mesoendemic areas [17,19,41].

One should be aware that a similar profile of IgG, IgG-isotypes and IgM antibody responses to the OR domain was apparent in the Candeias do Jamari -1993 and Peixoto de Azevedo groups, both exhibiting a predominance of IgG1 and IgG3 OR specific antibodies together with both cytophilic and complement fixing isotypes, in accordance with the data recently reported for the Uganda population [18]. However, in Candeias do Jamari, from 1993 to 2002, there was a distinct pattern of antibody response to the P126 OR domain. In Candeis do Jamari 1993, IgG1 and IgG3 P126 OR responders were predominant whereas in 2002 the predominant subclasses were IgG3 and IgG4. However, in 2002 the frequency of IgM and IgG2 responders increased while IgG1 and IgG3 decreased in comparison with 1993, although antibody levels were similar in both groups excluding IgG1 which was significantly lower in 2002. Therefore, it was herein demonstrated that P126 OR domain elicits IgG3 antibody response polarization in each group regardless of the infection patterns. The IgG1 and IG3 subclasses are thought to be involved in antimalarial immunity mediated by monocytes through antibody-dependent cytotoxic inhibition. In this respect, IgG1 and IgG3 antibodies are the predominant IgG subclass responses to the OR domain and are associated with protection in man living in malaria-endemic areas [16,17]. The marked switch of anti-OR IgM and IgG isotype patterns present in the Candeias do Jamari groups from 1993 to 2002 is probably related to the decrease of parasite density from 9,642 in 1993 to 2,150 parasites/μL in 2002, the decrease of the API from 549 in 1993 to 173 in 2002 and increased cumulative exposure time to malaria infection from 13 to 21 years. Moreover, the Peixoto de Azevedo group as well as Candeias do Jamari-1993 presented high parasite density (9,288 and 9,642 parasites/μL, respectively), API (292 and 549, respectively), short cumulative exposure time to malaria infection (4 and 13 years, respectively) and similar IgG, IgM and IgG isopype patterns of anti-OR antibodies. These findings are in line with previous reports [42,43] that have shown an increase of both IgG1 and IgG3 prevalence associated with increase in malaria transmission and parasite load. These data suggest that individual exposed to malaria infections for a shorter period of time (Candeias do Jamari -1993 and Peixoto de Azevedo) develop little acquired immunity, consequently presenting high parasitaemia levels which could boost the IgG1 and IgG3 response and prevalence. In contrast, individuals exposed to malaria infection for a longer period of time (Candeias do Jamari -2002), develop some level of acquired immunity, consequently displaying low parasitaemia which may not be able to enhance the antibody subclass responses. If IgG1 and IgG3, specific to the OR-domain, participate in the protection, as declared in several studies of the immune response to *P. falciparum *vaccine candidates [17,42,44-47], the parasite levels may modulate the induction of antibody subclass responses necessary to control parasitaemia through differential catabolism and/or use of these antibodies.

One should be aware that the prevalence of antibody responses to the OR domain in the Peixoto de Azevedo group infected with different patterns of the *P. falcipar*um P126 OR domain presented a similar profile of IgG, IgG subclasses and IgM antibody response to the OR domain. Therefore, infection with *P. falciparum *containing OR-I and/or OR-II does not influence the response to the OR-II domain suggesting cross-reactivity of two epitopes within the repetitive sequence. Indeed, ELISA competition assay has already demonstrated that human sera from a population exposed to malaria recognize OR domains containing a minimum of four repeats [17].

## Conclusion

In conclusion, there is a display of a limited genetic polymorphism of the P126 OR domain in *P. falciparum *isolates in a Brazilian population. This polymorphism does not seem to influence the development of a specific humoral immune response (quality and immunogenicity), which is paramount considering that genetic polymorphism, even though limited in regions encoding immunodominant epitopes, is important for malaria vaccine development regarding the potential parasite escape mechanisms involved with respect to the host immune system. Thus in addition, it was confirmed that there was the predominance of cytophilic antibodies against the P126 OR domain could participate in cellular antibody cooperation mechanisms, which might play a prime role in the development of protective immune responses against *P. falciparum *malaria infection [17,42,47]. Finally, the P126 OR domain could be included as a component of a multi-valent malaria vaccine.

## Competing interests

The authors declare that they have no competing interests.

## Authors' contributions

LRLR participated in the design of the study; carried out the molecular and immunological studies; performed the statistical analysis and drafted the manuscript, SLSS participated in the design of the study and helped in molecular analysis, BTS helped in immunological analysis, MLG and MGM helped in molecular analysis, FS and JOF helped in design and implementation of field studied, MGZ, DC, MFFC and CTDR helped in the design of the study, TSS and GSS helped in predictive analysis, DMB conceived of the study, and participated in its design and coordination and helped to draft the manuscript. All authors have read and approved the final manuscript.
